# Mechanisms Underlying Food-Triggered Symptoms in Disorders of Gut-Brain Interactions

**DOI:** 10.14309/ajg.0000000000001812

**Published:** 2022-05-04

**Authors:** Karen Van den Houte, Premysl Bercik, Magnus Simren, Jan Tack, Stephen Vanner

**Affiliations:** 1 Translational Research Center for Gastrointestinal Diseases, University of Leuven, Leuven, Belgium;; 2 Department of Medicine, Farncombe Digestive Health Research Institute, McMaster University, Hamilton, Ontario, Canada;; 3 Department of Molecular and Clinical Medicine, Institute of Medicine, Sahlgrenska Academy, University of Gothenburg, Gothenburg, Sweden;; 4 Gastrointestinal Diseases Research Unit, Queen's University, Kingston General Hospital, Kingston, Ontario, Canada.

## Abstract

There has been a dramatic increase in clinical studies examining the relationship between disorders of gut-brain interactions and symptoms evoked by food ingestion in the upper and lower gastrointestinal tract, but study design is challenging to verify valid endpoints. Consequently, mechanistic studies demonstrating biological relevance, biomarkers and novel therapeutic targets are greatly needed. This review highlights emerging mechanisms related to nutrient sensing and tasting, maldigestion, physical effects with underlying visceral hypersensitivity, allergy and immune mechanisms, food–microbiota interactions and gut-brain signaling, with a focus on patients with functional dyspepsia and irritable bowel syndrome. Many patients suffering from disorders of gut-brain interactions exhibit these mechanism(s) but which ones and which specific properties may vary widely from patient to patient. Thus, in addition to identifying these mechanisms and the need for further studies, biomarkers and novel therapeutic targets are identified that could enable enriched patient groups to be studied in future clinical trials examining the role of food in the generation of gut and non-gut symptoms.

## INTRODUCTION

A large proportion of patients suffering from disorders of gut-brain interactions (DGBIs) report that the ingestion of food triggers symptoms, but this relationship had received little attention for many years. However, this has changed dramatically in the past decade with a dramatic increase in published studies, particularly those examining functional dyspepsia (FD) and irritable bowel syndrome (IBS). Collectively, clinical studies overwhelmingly support that dietary factors can strongly influence DGBI symptoms, yet their interpretation is often complicated by the challenges of conducting dietary studies. These include patient and researcher bias, difficulties in blinding, placebo and nocebo responses, and low-quality study design. Moreover, an inability to identify patients where a diet is mechanistically implicated could lead to many patients enroled in studies that would ultimately not benefit and inclusion of these patients in the analysis could potentially dilute out a positive benefit of the dietary intervention. Thus, understanding mechanisms are critical to: (i) validating the results of clinical trials, (ii) identifying potential biomarkers to enrich treatment groups, and (iii) providing novel treatment therapies that might avoid the inconvenience and possible detrimental effects of restrictive diets. To address this need, we examine current mechanisms being explored, possible biomarkers and identifies knowledge gaps for future studies. The main focus is on FD and IBS as mechanistic studies have largely examined patients with these disorders to date.

## Nutrient sensing and tasting

**Figure 1.**
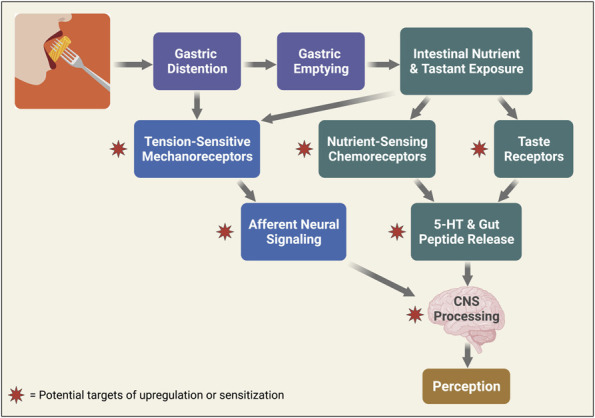
Sequence of physiological events related to the presence and sensing of nutrients in the gastrointestinal tract. Potential sites of upregulation or sensitization leading to visceral hypersensitivity are indicated by red stars. Created with BioRender.com. CNS, central nervous system.

The gastrointestinal (GI) tract displays 3 types of sensory modalities: mechanosensitivity, chemosensitivity, and thermosensitivity—all of which can be activated by the ingestion of food and can contribute to gut-brain signals controlling food intake (Figure 1) (1). The role of thermosensitivity is most likely limited, although cold meals may empty more slowly from the stomach (2). The current view is that gastric sensing of the presence of food is mainly mechanosensitive, volumetric sensing, while chemosensing of nutrient composition occurs in the small bowel (1,3). The volume of the meal is sensed by gastric mechanoreceptors, and the available evidence suggests that these behave like tension-sensitive in-series mechanoreceptors (4). Their molecular identity and precise location have not been determined. Mechanosensitivity to luminal distention is also a feature of the small bowel and the colon (5), and like the stomach is likely to be influenced by food quantity, composition, and non-digestible residue (6).

In terms of nutrient chemosensing, the upper small bowel expresses a large number of specialized receptors which can detect all the main categories of nutrients. The presence of glucose, fatty acids, and amino acids is monitored through large numbers of specific receptors or transporters (1,7,8). These include direct depolarisation through the sodium-glucose co-transporter-1 (SGLT1); G-protein-coupled receptors, FFAR1 (previously GPR40), FFAR3 (previously GPR41), FFAR2 (previously GPR43) and FFAR4 (previously GPR120); CD36; the calcium-sensing receptor; metabotropic glutamate receptors, GPRC6A and GPR93. Activation of these receptors leads to release of gut peptides and mediators such as cholecystokinin (CCK), glucagon-like peptide 1 (GLP-1), GPCR interacting protein, peptide YY (PYY), 5-hydroxytryptamine (5-HT) and the lipid signaling molecule oleoylethanolamide (1,7,8).

Besides nutrient sensors, the gut mucosa expresses transient receptor potential channels, which serve as receptors for a number of tastants. Examples are the TRPV1 receptor for capsaicin, the TRPM8 receptor for menthol and the TRPA1 receptor for cinnamaldehyde (8,9). The mucosa also expresses taste receptors of the G-protein coupled families T1R and T2R which are sensitive to stimuli similar to taste cells on the tongue. Taste receptors and several TRP channels seem to be mainly expressed on entero-endocrine cells, where they also modulate the release of gut peptides in response to presence or absence of nutrients in the lumen (1,7,8,10).

Several DGBIs are characterized by or associated with alterations in these sensory processes. Enhanced nutrient sensing of lipids has been reported in FD, where duodenal lipid exposure leads to enhanced release of CCK, altered gastric sensorimotor control and increased postprandial symptoms. These events are in part inhibited by a lipase inhibitor, orlistat, or by the CCK receptor antagonist dexloxiglumide (11–14). In addition, exaggerated release of gut peptides in response to intraduodenal administration of lipids and glucose has been reported in FD patients (15). Whether such alterations also occur upon normal oral food intake has not been reported so far. In IBS, increased release of 5-HT in response to nutrient ingestion has been reported in diarrhea-predominant IBS (IBS-D), and decreased release in constipation-predominant IBS (IBS-C) (16,17).

In FD, increased sensitivity to capsaicin administration has been reported, suggesting hypersensitivity to TRPV1 receptor activation (18). In healthy subjects, bitter tastants inhibit the release of motilin and ghrelin and suppress the occurrence of gastric phase 3, which is associated with decreased hunger sensations (19,20), but alterations in DGBI have not been studied to date. Low- or non-caloric sweeteners such as erythritol and xylitol do not alter plasma glucose or insulin levels, can stimulate the secretion of gut peptides such as CCK, GLP-1 and PYY, and slow down gastric emptying (21,22). Whether these responses are altered in DGBI patients has also not been addressed to date.

## Defective processing

Most of the available evidence suggests no alteration in digestion or absorption of nutrients in patients with DGBIs compared to the general population. However, well-known and widely available maldigestion/malabsorption syndromes may more easily lead to symptoms in subjects with DGBIs, presumably related to abnormal sensorimotor function in the GI tract and abnormal gut-brain interactions (23,24).

Lactose malabsorption is a well established and common entity that might lead to symptoms such as abdominal pain, bloating and diarrhea after lactose ingestion, i.e. lactose intolerance (25). The disaccharide lactose cannot be absorbed, but has to be cleaved by the small intestinal brush border enzyme lactase-phlorizin into the monosaccharides glucose and galactose which are then actively transported into the enterocytes by the sodium (+)/glucose (galactose) cotransporter (SGLT1) (26). Inability to digest lactose properly because of lactase non-persistence, leading to lactose malabsorption, is prevalent and found in 68% of subjects worldwide with large differences across the world with the lowest prevalence in Nordic countries (<5% in Denmark) and close to 100% prevalence in Korean and Han Chinese populations (27). Even though lactose malabsorption due to lactase non-persistence does not appear to be more common in patients with DGBIs, lactose intolerance may be more common in IBS (28) and related to visceral hypersensitivity, immune alterations in the gut and psychological factors, which point towards the relevance of gut-brain interactions (29).

The involvement of fructose malabsorption in the generation of symptoms in patients with DGBIs has also received significant attention recently (30). Fructose is a monosaccharide that is slowly absorbed in the small intestine by carrier-mediated facilitated diffusion, predominantly via 2 fructose carriers belonging to the glucose transport (GLUT) family of sugar transporters, GLUT5 and GLUT2. GLUT5 is specific for fructose movement, and low-affinity and concentration dependent, whereas GLUT2 is a high-capacity pathway for the absorption of glucose, galactose, and fructose. Fructose absorption via GLUT2 is facilitated by a high glucose concentration. Hence, the luminal fructose concentration, but also the amount of glucose, influence fructose absorption (31). Malabsorption of 25 g of fructose can be identified in approximately 20% of patients with IBS (30), which may be higher than in control populations. However, the mechanisms underlying symptom generation after fructose ingestion, i.e. fructose intolerance, is not completely understood, and does not seem to be related primarily to the degree of malabsorption, but rather to microbiota composition and function, gas production, and visceral sensation (32–34).

Congenital sucrase-isomaltase deficiency is an inherited deficiency in the ability to hydrolyze sucrose, maltose, short 1–4 linked glucose oligomers, branched (1–6 linked) α-limit dextrins, and starch. Exposure to these nutrients in affected patients provokes osmotic diarrhea with pain, bloating, and abdominal distention (35). In addition to the congenital form, acquired or secondary forms of sucrase-isomaltase deficiency have been observed in patients with chronic diarrhea, and may be the more commonly encountered problem in adults (36,37). Recently, several publications reported an increased prevalence of hypomorphic (defective) sucrase-isomaltase (SI) gene variants in IBS, with a link to IBS with diarrhea (38–40). The reduced SI enzymatic activity may trigger IBS symptoms via colonic accumulation of undigested disaccharides from starch and sucrose, resulting in fermentation with gas production and osmotic diarrhea. Patients with reduced SI activity as a contributing abnormality to their symptoms may therefore be less likely to respond favorably to dietary treatment approaches not focusing on reducing starch and sucrose in the diet, e.g., a low fermentable oligosaccharides, disaccharides, monosaccharides and polyol (FODMAP) diet or traditional IBS dietary advice, as suggested in a recent post-hoc analysis of a dietary intervention trial (41). Hence, considering reduced SI enzyme activity in patients with IBS (in particular the diarrheal subtype) should be considered when patients with meal-related symptoms do not respond favorably to standard dietary approaches, and sucrose and starch reduction may then be tested (42).

## Physical effects with underlying visceral hypersensitivity

DGBI disorders express combinations of a number of pathophysiological alterations, including motility disturbances, visceral hypersensitivity, altered mucosal and immune function, altered gut microbiota, and altered central nervous system processing (43). These pathophysiological processes are central for food-induced symptoms, and as noted in the previous paragraph, visceral hypersensitivity, gut microbiota composition and function, and central nervous system function were identified as key mechanisms explaining symptoms in IBS with carbohydrate malabsorption. Both fructose (in excess of glucose) and lactose are included in the FODMAP concept, which includes carbohydrates that are incompletely absorbed in the small intestine owing to absent hydrolysis (e.g., lactose malabsorption or nondigestible oligosaccharides), dependence on simultaneous intake of glucose for adequate absorption (fructose) or passive diffusion (certain monosaccharides and polyols). In addition, their absorption depends on different factors such as small intestinal transit time, dose of the carbohydrate, meal composition and the presence or absence of mucosal disease (44). The incompletely absorbed short-chain carbohydrates that pass into the large intestine are fermented by gut bacteria, leading to gas production and intestinal distention. Moreover, through osmosis, there is a net flux of water into the lumen, and FODMAPs can also stimulate motility (45). Together, all these mechanisms might contribute to symptom generation in susceptible individuals, through different mechanisms. In line with the proposed symptom generating mechanisms in DGBI subjects with lactose and fructose malabsorption, visceral hypersensitivity and gut-brain interactions seem to be of importance in symptom generation after intake of food rich in FODMAPs (46) in many patients with DGBI. The central role for visceral hypersensitivity and gut-brain interactions in symptom experience after intake of FODMAPs was supported by a study measuring gas production, intestinal content and volume, and symptoms after FODMAP intake in IBS and controls (Figure 3). Patients reported more severe symptoms, but the physiological responses in terms of gas production and intestinal content were similar in patients and controls, which suggests that visceral hypersensitivity rather than excessive gas production is the main driver for carbohydrate-related symptoms in patients with IBS (34). However, there are other studies that use magnetic resonance imaging (MRI), suggesting that subtypes of IBS may respond differently to meal ingestion than healthy controls, potentially relating to IBS subtype-specific differences in transit and intestinal tone, as well as in secretion and absorption (47,48) and microbiota, as outlined below. Furthermore, distinct abnormalities of small bowel and regional colonic volumes have been revealed by MRI in IBS subtypes (49), which may relate to different symptom responses to meal ingestion.

Altered GI motility is a key pathophysiological factor in IBS and other DGBIs and has partly guided drug development for these disorders (50). Abnormal GI motor responses to meal intake and specific nutrients in IBS and other DGBIs have been frequently reported. Examples of this are an exaggerated and prolonged gastrocolonic motor response in IBS (51–53), and exaggerated colonic motility with higher frequency and amplitude of high amplitude propagated contractions and higher motility index in IBS-D, in particular in response to a meal and associated with pain reports (54). *In vitro* experiments demonstrated that this exaggerated response was suppressed by both a CCK antagonist and atropine, suggestive of disordered enteric nervous system function explaining this abnormality (54). Furthermore, as stated above, MRI has revealed differences in intestinal responses to meal intake in IBS subjects, related to changes in intestinal transit, tone, secretion and absorption (47,48). Hence abnormal motor responses to meal ingestion in patients with IBS and other functional bowel disorders seem to be of relevance for meal-related symptoms in this group of patients.

Not only various carbohydrates, but also fatty foods commonly trigger symptoms in IBS and other DGBIs (55,56), This can partly be explained by exaggerated physiological responses in the GI tract in patients with DGBIs. For example, the fat component of the diet is the predominant stimulus of colonic motor activity in response to eating (57–59), and as stated previously this response is exaggerated in IBS (51–53). Furthermore, the motor dysfunction demonstrated in patients with IBS can also result in gas retention within the gut and the appearance of symptoms (60), and physiologic concentrations of intestinal lipids inhibit intestinal gas transit, a mechanism that is up-regulated in some patients with IBS (61). This strengthens the role of abnormal GI motor responses to lipids in IBS being of importance in the symptom generation after fatty foods. Additionally, visceral hypersensitivity, considered to be one of the key pathophysiological mechanisms in various DGBI (62), is enhanced after administration of duodenal lipids in patients with IBS, suggestive of an enhanced sensory component of the gastrocolonic response (63,64). Hence, several abnormal physiological responses to lipids in the GI tract are likely of importance for meal-related symptoms in these patients.

## ALLERGIC AND IMMUNE MECHANISMS

### Non-celiac gluten sensitivity

Non-celiac wheat sensitivity (NCWS), or non-celiac gluten sensitivity, manifests with both GI and extra-intestinal symptoms after ingestion of wheat. Despite its high reported prevalence, which varies between 0.6% and 13%, the pathophysiology of NCWS is poorly understood (65). Apart from gluten, other wheat components such α-amylase-trypsin inhibitors, known to induce low-grade gut inflammation through the TLR-4 signaling, and fructans, part of FODMAP group, were proposed as symptom triggers (66). There are no established biomarkers of NCWS, although some studies suggest that anti-gliadin immunoglobulin G may help to identify those patients who benefit from wheat/gluten restriction (67,68).

### GI-specific immune activation or intolerances

#### Upper GI tract.

**Figure 2.**
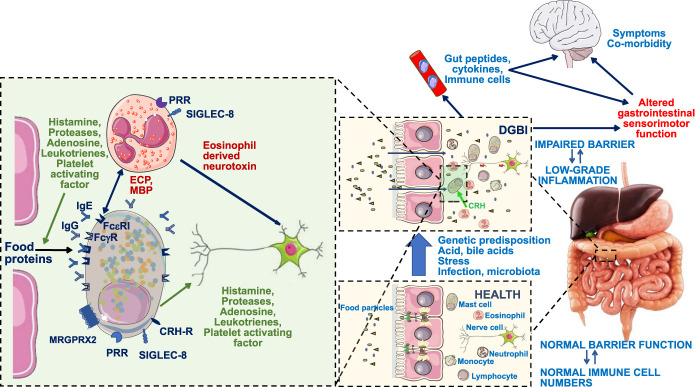
Hypothetical mechanisms involved in allergy-like reactions to food in the gastrointestinal tract in disorders of gut-brain interactions (DGBIs), as hypothesized for food reactions mainly in irritable bowel syndrome (IBS) and functional dyspepsia (FD). Increased mucosal permeability is proposed as an underlying alteration, allowing food proteins in the lumen to activate mast cells and/or eosinophils in DGBI patients. The latter may lead to release of cytokines and other signaling molecules in the circulation, recruitment of inflammatory cells and altered neural (e.g., through eosinophil-derived neurotoxin) and hormonal control of gastrointestinal sensorimotor function, triggering nutrient-induced symptoms. The mechanism through which food proteins activate mast cells or eosinophils in DGBIs remains to be established. Proposed pathways involve locally produced immunoglobulin E (IgE) acting on the FceR receptor, immunoglobulin G (IgG) acting on the FcgR receptor or non-Ig mediated activation of mast cells through pattern recognition receptors (PRRs) or the mas-related G-protein coupled receptor X2 (MRGPRX2). Mast cells can also be activated by corticotrophin releasing hormone (CRH). The submucosal inflammatory cells can be inactivated through sialic acid-binding immunoglobulin-type lectin (SIGLEC) receptors. Further studies will be required to identify the contribution of these putative pathways in (subgroups of) specific DGBIs.

Recent emerging evidence indicates a role for food-induced mucosal alterations in the pathogenesis of upper GI alterations and symptoms. A number of studies used confocal laser endomicroscopy to demonstrate duodenal mucosal reactions to food allergens in patients reported to have IBS (69–72). In a preliminary report, improvement of symptoms and duodenal barrier function was observed after 6 weeks of a low FODMAP diet in FD (73). The traditional view on the mechanism of action of FODMAPs, i.e., osmotic activity and fermentation by gut microbiota, cannot readily explain these mucosal events, which are in line with changes in urinary histamine content during the low FODMAP diet in IBS (74). The most recent research has focused on local mucosal responses to food proteins (Figure 2). Pilot studies showed acute reactive changes in the duodenal mucosa detected by confocal laser endomicroscopy upon application of food proteins, and improvement of symptoms upon elimination of 6 allergenic foods (soy, wheat, milk, egg, nuts, fish/shellfish) in FD patients (75,76). A likely prerequisite for duodenal reactions to mucosally applied nutrients is increased mucosal permeability (77,78). In both the confocal laser endomicroscopy and in the 6 food elimination diet studies, similar to the IBS studies, patients were negative on blood tests for classical immunoglobulin E (IgE)-mediated food allergies (69–72,75,76). One hypothesis is local production of food-targeting IgEs (79), but to date there are no reports that have evaluated this possibility in the upper GI tract. A potential role for food immunoglobulin G antibodies remains an area of controversy (68,80–82). A second hypothesis is activation of mast cells through non-IgE-mediated pathways. Emerging evidence points toward enhanced expression and activity of the Mas-related G-protein coupled receptor X2 in the duodenum of patients fulfilling FD characteristics (83). Future research will be needed to identify the actual pathway, which is likely to lead to novel treatment approaches, besides individualized dietary elimination.

#### Lower GI tract.

**Figure 3.**
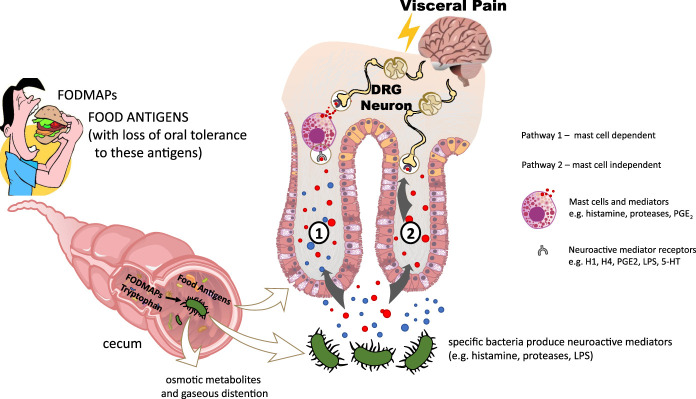
Food evokes and amplifies visceral hypersensitivity via mast cell dependent (**1**) and mast cell-independent pathways (**2**). Ingestion of high FODMAP foods (and other poorly absorbed sugars) leads to bacterial fermentation predominantly in the colon, producing gas and osmotically active metabolites that can distend the colon and amplify pre-existing visceral hypersensitivity. Specific bacteria also produce neuroactive mediators that evoke or amplify visceral hypersensitivity via mast cell dependent and independent pathways. Intermediary cells (e.g., enterocytes, enteroendocrine cells or other immune cells) may also contribute to either pathway. Re-exposure to food antigens following loss of oral tolerance caused by an acute self-limiting infectious colitis or psychological stress could also lead to IgE-dependent mast cell activation and visceral hypersensitivity. 5-HT, 5-hydroxytryptamine; FODMAP, fermentable oligosaccharides, disaccharides, monosaccharides and polyols; LPS, lipopolysaccharide.

The landmark discovery that mast cells are activated in the colon and small bowel of IBS patients, that they are closely associated with intestinal nerves and their mediators, particularly histamine and proteases, and that their activation causes visceral hypersensitivity (84–86) has directly implicated the immune system in IBS pathophysiology (Figure 3). Moreover, it posed the question, “What is the mechanism(s) leading to their activation?” While multiple mechanisms are likely involved, recently studies (79) suggest food can invoke a local immune response within the intestine leading to mast cell activation.

Systemic food allergy had been implicated and while careful clinical testing ruled out this cause, clinical clues suggested that food could cause a local immune response in IBS patients. For example, injection of food allergens into the cecum of patients with food sensitivities, evoked a local weal and flare reaction despite low levels of serum IgE for those antigens (87) and studies utilizing confocal laser endomicroscopy showed that perfusion of food antigens into the duodenum resulted in increased intraepithelial lymphocytes and widened intervillous spaces in the majority of IBS patients with suspected food intolerances compared to healthy volunteers (69). This concept of a localized immune response had also been described in the nose, where local allergic rhinitis occurs with mast cell activation in the nasal mucosa (88).

Recent novel studies provided direct evidence by demonstrating that common IBS triggers can break oral tolerance to food antigens (79,89). Self-limiting infectious colitis has long been recognized as a trigger for IBS and recent preclinical studies demonstrate self-limiting bacterial colitis breaks oral tolerance to food antigens and that re-exposure to food antigen causes mast cell activation and visceral hypersensitivity in the colon (79). In the same study, injection of common food antigens into the rectal mucosa caused wheal and flare in patients with IBS but not controls. Mucosal biopsies show that mast cell IgE immunochemical staining is greater in patients with IBS than healthy controls. Preliminary studies suggest psychological stress can also break oral tolerance to food antigens (89), and re-exposure leads to mast cell activation in both the colon and small intestine and ultimately, visceral hypersensitivity. Whether a Th2 paradigm underlies this mechanism is unclear but evidence of a STAT-6-dependent mechanism in preliminary studies is supportive.

## Microbiota: food interactions

The compelling evidence that lowering dietary FODMAPs in IBS patients significantly reduces abdominal pain and bloating has provided a strong rationale to examine underlying mechanisms (90). While colonic fermentation of these poorly absorbed carbohydrates produces gas and abdominal distention that could aggravate preexisting visceral hypersensitivity (34), emerging studies suggests the diet-microbiota interactions are a critical source of neuroactive mediators that significantly modulate intestinal nociceptive signaling and cause visceral hypersensitivity (91–94).

Multiple bacterial mediators have been implicated, including histamine, proteases, tryptamine, 5-HT, and lipopolysaccharide (91–98). In addition to FODMAPs, gut microbial composition has been implicated in dietary tryptophan metabolism and microbial metabolites can module the gut-brain axis (96–98). A proportion of dietary tryptophan reaches the large intestine where its metabolism by commensal microbes producing a number of neuroactive mediators including 5-HT, tryptamine and indols that have been implicated in IBS (Gao). More direct evidence comes from studies of the actions of fecal supernatants from IBS patients that were compared to healthy controls by perfusing them into *ex vivo* mouse colons and recording mechanosensitivity of afferent nerves. These studies show that IBS fecal supernatants signaling from the lumen to nerve terminals in the intestinal wall cause visceral hypersensitivity. This effect was blocked by histamine antagonists in some patients and proteases inhibitors in others. Importantly, following a low FODMAP diet in these patients this visceral hypersensitivity action was lost. Histamine has also been directly implicated in combined clinical and reverse translational studies where abdominal pain and urinary histamine levels decreased in a subset of patients after lowering intake of these carbohydrates (74). To investigate the role of the microbiota in this abdominal pain, IBS microbiota from study patients were colonized in germ-free mice, creating a “humanized IBS model” (94). Using fecal microbiota from IBS patients exhibiting high urine histamine (HH) and high pain following FODMAPs and low urine histamine and low pain response (LH) following FODMAPs patients in this model, a histamine-dependent microbial pathway causing visceral hypersensitivity was identified in the HH patients. Together, these studies demonstrate a food-microbiota interaction can produce multiple neuroactive mediators and that these may vary between patients.

The evidence that production of neuroactive mediators based on food-microbiota interactions may be individualized suggests that corresponding differences in microbiota may underlie these findings (94,99–101). This relationship has been most carefully studied for histamine to date (94), which can be produced by many bacteria through the activity of histidine decarboxylase (*HDC*), a gene that can be found in 2 different isoforms in both Gram- and Gram+ bacteria, but is far more efficient in Gram- Proteobacteria. *Klebsiella aerogenes* was identified as the main histamine producer in IBS-HH microbiota, producing >100× more histamine than any other bacterial isolate. *K. aerogenes* is higher in IBS-HH patients and its specific *HDC* gene is present in the stool in many but not all IBS patients. Together, these data strongly suggest that in a subgroup of IBS patients, specific microbiota are a driver of visceral hypersensitivity that is dependent on histamine signaling. Further studies are needed to identify specific bacteria or communities that produce other neuroactive mediators and how specific foods influence their production.

There is also evidence that the microbial derived neuroactive mediators resulting from food-microbial interactions can sensitize nociceptive nerves directly or indirectly, including via mast cell dependent pathways. Histamine signaling likely involves H1 and H4-receptor dependent pathways (94) and lipopolysaccharide activates mast cell dependent PGE_2_ signaling to nociceptive nerves (91,92). Thus, food-microbial interactions are likely one cause of the mast cell activation that has been widely recognized in IBS patients for the past several decades. Most studies have largely studied IBS-D patients and further studies are needed to determine whether food-microbial interactions also play a role in pain related to other IBS subtypes, such as IBS-C.

## Gut-brain interactions

The GI tract communicates with the brain in a bidirectional fashion through neural, immune and hormonal pathways. This communication, which is generally referred to as the “gut-brain axis,” constitutes a core part of the integrated interoceptive system through which information about the body's physiological condition is continuously transmitted to the brain (102), and which assures proper maintenance of GI homeostasis and digestion. Gut-brain axis signaling has effects on mood, motivation, and higher cognitive functions, and in turn is affected by stress, anxiety, and depression. Gut microbiota plays a key role in gut-brain axis signaling, as bacteria can communicate with the host through molecules with neuroactive or immunomodulatory properties, that result from bacterial metabolism of dietary components (103).

**Figure 4.**
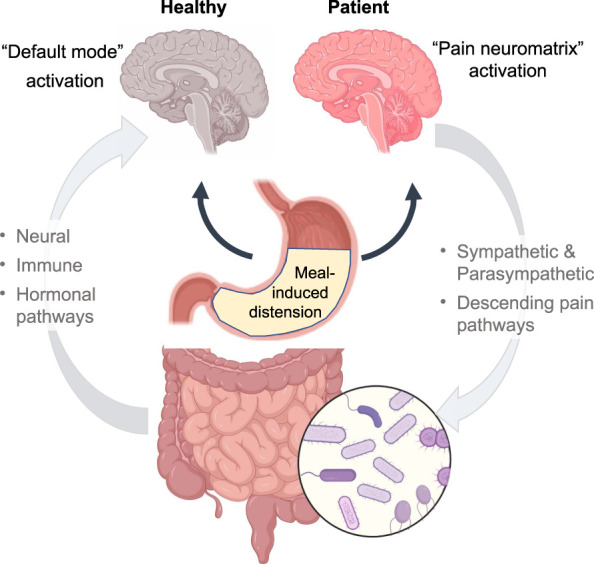
A hypothetical central mechanism to explain different responses to meals in healthy individuals and patients with functional gastrointestinal disorders. Gastrointestinal tract communicates with the brain through neural, immune and hormonal pathways, and the microbiota plays a key role in this gut-brain axis. While in healthy subjects, meal induced distension activates “default mode network” in the midbrain, in patients the gut distension activates pain-responsive regions.

The gut-brain axis has a major role in the perception of unpleasant or nociceptive stimuli arising from the digestive tract, caused by gut distension, chemical stimuli or inflammation, allowing to discriminate between these modalities. A study in healthy volunteers demonstrated that brain responses to gradual gastric distension induced by a balloon or a liquid meal containing proteins, carbohydrates and lipids, differed despite achieving similar levels of distension. While the balloon distension progressively activated pain-responsive regions (pain neuromatrix), nutrient infusion deactivated the pain neuromatrix while activating the “default mode network” in the midbrain, which was associated with changes in the plasma levels of ghrelin and PYY (Figure 4) (104). These differential brain responses may constitute the neurophysiological mechanism underlying the tolerance of normal meal volumes in healthy individuals, and their impairment may explain symptom genesis in patients with food-triggered symptoms.

## SUMMARY, EMERGING BIOMARKERS AND THERAPIES, AND KEY AREAS OF FUTURE STUDY

This review highlights the emerging evidence that specific mechanisms underlie food-induced symptoms in patients suffering from DGBI, and highlights that most of the work has focused on FD and IBS (Figure 1). Increasingly the evidence shows that these mechanisms are found in a select but significant number of DGBI patients. It is also important to understand that these mechanisms are not mutually exclusive, and, indeed, are very likely to overlap in many patients. From these mechanistic studies a list of putative biomarkers and new therapeutic targets can be constructed (Table 1). To our knowledge, none of the mechanistic studies have used visceral hypersensitivity as an entry criteria for IBS studies. The same is true for FD studies. The question whether treatments targeted at visceral hypersensitivity may improve symptoms in patients with normosensitivity remains to be addressed. There is extensive literature on the lack of good correlation between enhancement of gastric emptying and symptom improvement in FD with delayed emptying (105,106). The challenge going forward is to continue the discovery of novel mechanisms and to translate the findings into effective therapies. Given that many of these are personalized to individual patients, understanding the predictive value of specific biomarkers is needed to enrich clinical trials. To aid in reaching this goal, further studies of the different subtypes with FD and IBS are needed to determine if these symptom-based subclasses (e.g., IBS-D vs IBS-C) exhibit common and/or specific underlying pathophysiological mechanisms related to diet. Using the biomarkers, there are a number of intriguing food, pharmacological, and microbial therapies that can then be tested in these patients. Understanding which patients may benefit from novel pharmacological or microbial therapies could also overcome the concerns regarding the potential detrimental effects of restrictive diets including nutritional, microbial and psychological.

**Table 1.**
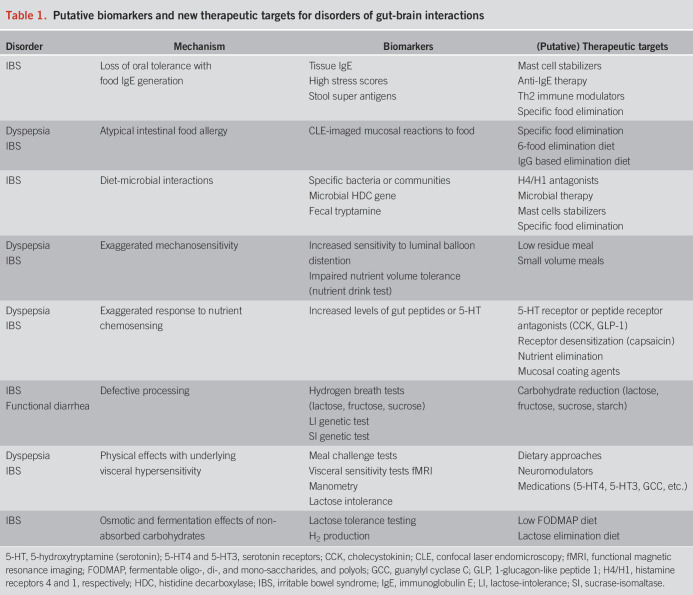
Putative biomarkers and new therapeutic targets for disorders of gut-brain interactions

## CONFLICTS OF INTEREST

**Guarantor of the article:** Stephen Vanner, MD, MSc.

**Specific author contributions:** All authors contributed equally to the writing of the manuscript and have approved the final submitted draft.

**Financial support:** S.V. is supported by Canadian Institutes of Health Research (CIHR) and Crohn's and Colitis Canada (CCC) operating grants.

**Potential competing interests:** None to report.
